# Physical activity, gestational weight gain in obese patients with early gestational diabetes and the perinatal outcome – a randomised–controlled trial

**DOI:** 10.1186/s12884-024-06296-3

**Published:** 2024-02-02

**Authors:** Lukasz Adamczak, Urszula Mantaj, Rafał Sibiak, Paweł Gutaj, Ewa Wender-Ozegowska

**Affiliations:** 1 Department of Reproduction, Chair of Fetomaternal Medicine, Poznan University of Medical Sciences, Poznan, Poland; 2 Doctoral School, Poznan University of Medical Sciences, Poznan, Poland; 3 Department of Histology and Embryology, Poznan University of Medical Sciences, Poznan, Poland

**Keywords:** Gestational diabetes mellitus, Physical activity, Obesity, Pregnancy

## Abstract

**Background:**

Excessive gestational weight gain, especially among women with gestational diabetes, is associated with several adverse perinatal outcomes. Our study aimed to analyse the impact of the use of pedometers to supervise physical activity on maternal health and the obstetric outcomes of pregnant women with obesity and early gestational diabetes.

**Methods:**

124 pregnant patients were enrolled in the presented research. Inclusion criteria: singleton pregnancy, age > 18 years, gestational diabetes diagnosed in the first half of pregnancy (< 20th week of pregnancy), obesity according to the American Endocrine Society criteria. Each patient was advised to take at least 5000 steps daily. Patients were randomly assigned to pedometers (*N* = 62), and were recommended to monitor daily the number of steps. The group without pedometers (*N* = 62) was not observed. Visit (V1) was scheduled between the 28th and 32nd gestational week (GW), and visit (V2) occurred between the 37th and 39th GW. Anthropometric measurements and blood samples were collected from all patients at each appointment. Foetal and maternal outcomes were analysed at the end of the study.

**Results:**

In the group supervised by pedometers, there were significantly fewer newborns with macrosomia (*p* = 0,03). Only 45% of patients satisfied the recommended physical activity guidelines. Patients who walked more than 5000 steps per day had significantly higher body weight at baseline (*p* = 0,005), but weight gain was significantly lower than in the group that did not exceed 5000 steps per day (*p* < 0,001). The perinatal outcome in the group of patients performing more than 5000 steps did not demonstrate significant differences with when compared to less active group. ROC curve for weight gain above the guidelines indicated a statistically substantial cut–off point for this group at the level of 4210 steps/day (*p* = 0.00001).

**Conclusions:**

Monitoring the activity of pregnant patients with gestational diabetes and obesity by pedometers did not have a significantly impact on their metabolic control and weight gain. However, it contributed to less macrosomia. Furthermore, physical activity over 5,000 steps per day positively affects weight loss, as well as contributes to improved obstetric and neonatal outcomes.

**Supplementary Information:**

The online version contains supplementary material available at 10.1186/s12884-024-06296-3.

## Introduction

Maternal obesity constitutes a significant risk factor for gestational diabetes mellitus (GDM), as well as other maternal and foetal complications. The risk of developing GDM in women with obesity or severe obesity is 4–8 times higher than in normal–weight women [1].

In the study by Pirkola et al., involving mothers with normal glucose tolerance, the prevalence of overweight offspring was 27.9% among overweight mothers, as compared to 13.5% among normal–weight mothers [2]. The abovementioned study shows that although hyperglycaemia increases the risk of offspring obesity, it is essential to address maternal weight, particularly if the mother suffers from overweight.

As the obesity among women of reproductive age grows, the primary goal of healthcare provider policy should be to reduce patients’ weight in these women before pregnancy and to strictly control gestational weight gain, because its impact on perinatal outcome is confirmed [3–6].

Physical activity and modification of dietary habits are considered the simplest steps towards weight control [7, 8], representing the key factors which allow to curb weight gain during pregnancy. The diet should be well-balanced and based on the healthy food pyramid [9]. In addition to commencing pregnancy at a healthy weight, women should also gain weight according to their BMI status during gestation. Notably, it was observed that the offspring of women with obesity, who had gained more weight than recommended during pregnancy, were at an increased risk of developing childhood obesity [10]. Thus, the introduction of programmes focusing on appropriate weight gain may positively affect the current and future pre–pregnancy maternal BMI. In fact, a meta–analysis of gestational weight gain programmes revealed that interventions promoting physical activity and providing dietary counselling, particularly when combined with weight monitoring, successfully reduced maternal gestational weight gain [11, 12].

Moreover, since 2009, when the Institute of Medicine (IOM) released the updated recommendations for gestational weight gain [13], several studies investigating the recommendations, in particular for women with obesity, have been conducted [14].

Excessive gestational weight gain, especially among women with gestational diabetes, is associated with several adverse perinatal outcomes, including impaired foetal growth, preterm deliveries, caesarean section, and hypertensive disorders of pregnancy, as well as newborn mortality and long–term metabolic disorders in the offspring [15, 16].

Multiple randomised controlled trials have evaluated the efficiency of lifestyle interventions on gestational weight gain. Although lifestyle interventions may alter gestational weight gain, they have not been found to improve perinatal outcomes. However, regular physical activity has been shown to reduce the percentage of pregnant women with gestational diabetes [17–19].

With the development of technology, programs, smartphone applications, and other devices, e.g. pedometers, have become increasingly employed to promote physical activity and encourage pregnant women to become more physically active [20, 21]. McCurdy showed in his study that weight loss during pregnancy in women with obesity has been found to be associated with decreased risks of macrosomia and caesarean delivery. However, a potential association with low foetal birth weight remains considered [22].

Women with gestational diabetes mellitus are instructed to monitor blood sugar and to adjust their dietary habits. In cases where, despite the efforts, hyperglycaemia is observed, insulin treatment needs to be introduced to maintain target glycaemic levels, which in turn may reduce the impact of hyperglycaemia on the subsequent adiposity in the offspring [23]. Children of women with untreated gestational diabetes mellitus show an increased risk of foetal macrosomia and other metabolic complications compared to both children of women with treated gestational diabetes mellitus and non–diabetic women. Furthermore, overweight and obese women with poorly controlled gestational diabetes mellitus, regardless of the treatment modality, present significantly higher rates of the composite outcome of metabolic complications, macrosomia, and large for gestational age (LGA), compared to women in all weight groups with well–controlled gestational diabetes mellitus [24]. Therefore, intensive insulin treatment and achieving reasonable glycaemic control in women with obesity and gestational diabetes mellitus may play a role in preventing adverse outcomes in the offspring.

Our study aimed to analyse the impact of the use of pedometers to supervise physical activity on maternal health and the obstetric outcomes of pregnant women with obesity and early gestational diabetes. Primary and secondary endpoints were determined and analysed.

In addition, it was also assessed whether the number of steps taken had an impact on maternal weight gain, metabolic results and neonatal outcomes evaluated to.

## Materials and methods

The study was conducted between 2018 and 2021 at the Department of Reproduction, Poznan University of Medical Sciences, Poland. From a total of 478 patients with GDM receiving care at our clinic, 124 pregnant patients were enrolled in the presented research. The study involved patients in a singleton pregnancy, age > 18 years, who had been diagnosed with gestational diabetes in the first half of pregnancy (< 20th week of pregnancy) and who satisfied the obesity criteria according to the American Endocrine Society [25]:

1. Grade 0 obesity: BMI ≥ 30 kg/m^2^, pre–pregnancy obesity, no history of: hypertension (HT), prediabetes, type 2 diabetes lipid disorders, non–alcoholic fatty liver, polycystic ovary syndrome, fertility disorders, sleep apnoea syndrome, asthma and gastroesophageal reflux;
2. Grade I obesity: BMI ≥ 25 kg/m^2^ and at least one mild or moderate abovementioned complication,
3. Grade II obesity: BMI of 25 kg/m^2^ and at least one severe complication listed above.

All pregnant women were diagnosed with gestational diabetes, if they satisfied one of the diagnostic criteria: fasting glycaemia ≥ 92 mg/dl (≥ 5.1mmol/l), glycaemia at 60 min oral glucose tolerance test (OGTT) ≥ 180 mg/dl (≥ 10mmol/l), glycaemia in 120, one minute OGTT ≥ 153 mg/dl (≥ 8.5mmol/L). Pregnancy hyperglycaemia was diagnosed according to the 2017 Polish Diabetes Association criteria, as well as to the IADPSG and WHO criteria [26, 27]. Of the 354 pregnant patients who were not included in the study: 276 had gestational diabetes diagnosed after the 20th week of pregnancy, 52 did not meet the obesity criteria according to the American Endocrine Society and 26 patients had multiple pregnancies. These patients did not fulfil the recruitment criteria of this study.

### Study design

During the first appointment, a diabetic diet was outlined to each patient, as well as the recommended caloric content of meals and weight gain during pregnancy, the conversion of carbohydrate exchangers, and the guided physical activity during pregnancy. According to the recommendations, each patient was advised to take at least 5000 steps daily. The lifestyle of this value [28]. The caloric demand was calculated individually for each patient following the guidelines of the Polish Diabetes Association and the American College of Obstetrics and Gynaecology, depending on initial body weight and BMI [26, 29]. Each patient was recommended to gain weight according to the guidelines of the International Federation of Gynaecology and Obstetrics, depending on the BMI before pregnancy, in order to achieve a weight gain of 5–7 kg [26].

The midwife responsible for education prepared and distributed sealed opaque envelopes containing the allocation, based on which the patients were assigned to the groups. She then informed the participants of the results of allocation. Pregnant women with odd study numbers received only the first diet and physical activity training (NP) (*N* = 62). In turn, pregnant women with even numbers received pedometers and the recommendation to supervise their physical activity by counting steps (P) (*N* = 62).

The primary endpoint was gestational weight gain (GWG) and changes in, maternal anthropometric parameters and HbA1C between the onset of observation and the V2 appointment (37 to 39 weeks). GWG was defined as the change in objectively measured weight from the baseline to the V2 visit, due to the fact that some women could not accurately provide their pre–pregnancy weight.

The secondary endpoint included neonatal birth weight, gestational age, as well as small–for–gestational–age (SGA) or large–for–gestational–age (LGA), and macrosomia.

The medical records data comprised comorbidities, obstetric and perinatal outcomes. In the course of the study, the authors analysed the incidence of neonatal complications, such as hyperbilirubinemia, hypoglycaemia, respiratory distress syndrome, and congenital malformations.

### Research protocol

Patients after GDM diagnosis (screening) were transferred to our department, where a research protocol consisting of three appointments was designed.

**Fig. 1:**
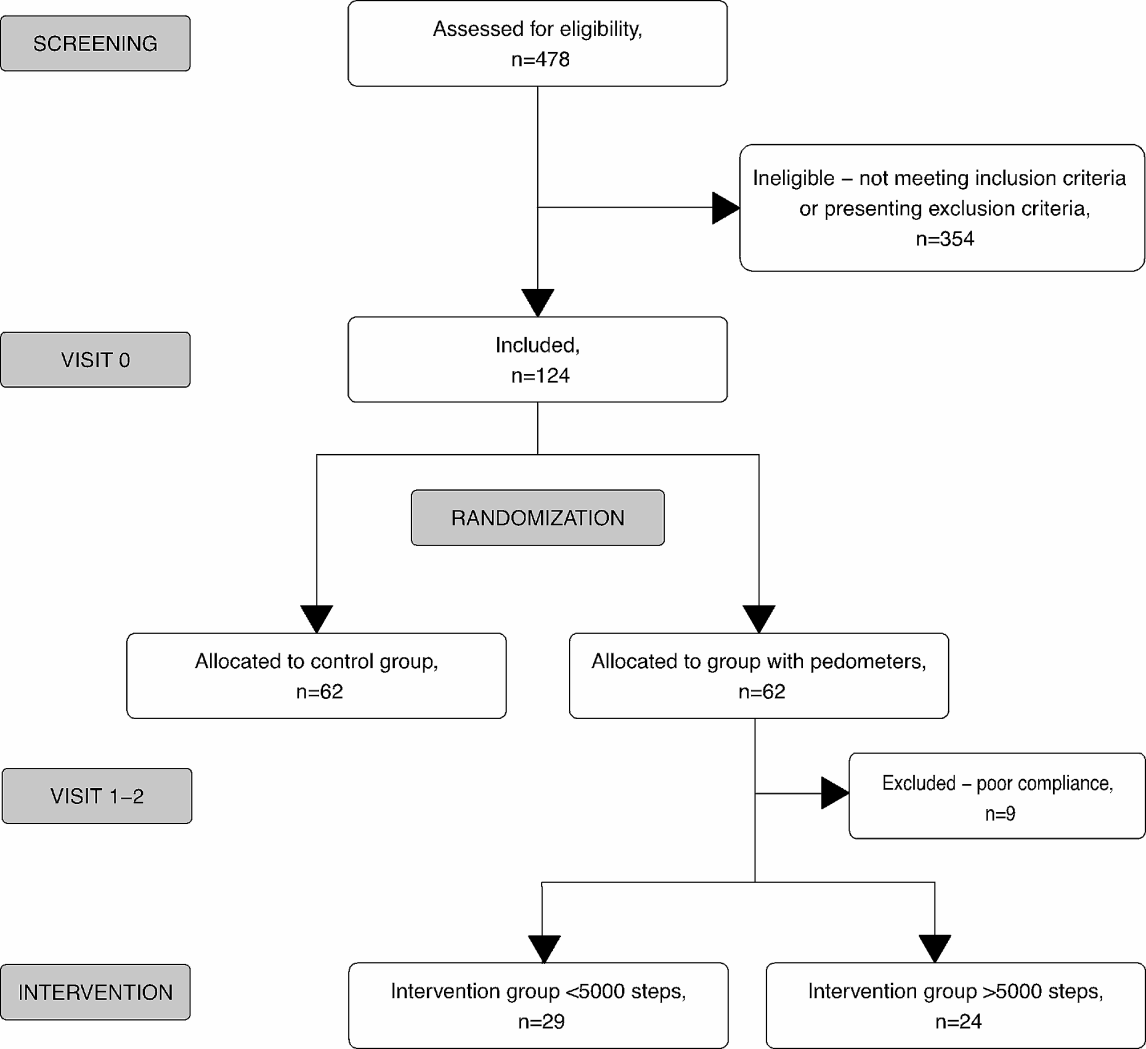
The course of the study

The whole GDM group treated in that period of time in our Department consisted of 478 GDM pregnant women. 124 pregnant women with obesity were recruited who met the inclusion criteria and consented to participate in the research were recruited (Fig. 1).

The enrolment visit (V0) occurred when the patient was first admitted to the department, immediately after diagnosing hyperglycaemia. During this appointment, patients were randomly assigned pedometers, and were recommended to monitor on a daily basis the number of steps. In contrast, the group without pedometers was not monitored for step count.

**Fig. 2:**
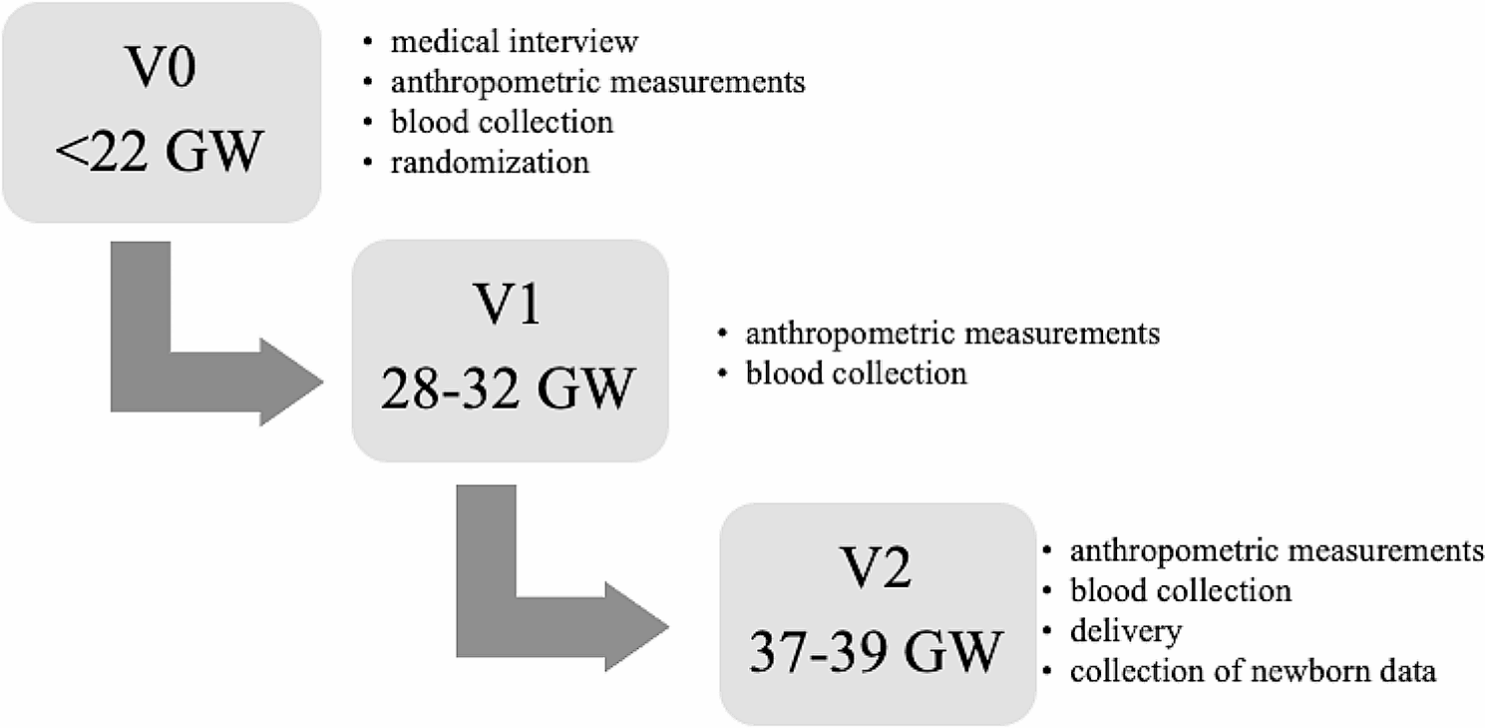
The study protocol

The first study visit (V1) was scheduled between the 28th and 32nd gestational week (GW), whereas the second study visit (V2) occurred between the 37th and 39th GW. Anthropometric measurements and blood samples were collected in all patients at each appointment. Foetal and maternal outcomes were analysed at the end of the study once the data regarding pregnancy, as well as obstetric and neonatal outcomes had been collected. Anthropometric measurements of newborns were estimated on the first day after birth (Fig. 2.).

Macrosomia was defined as a neonatal weight ≥ 4200 g in term pregnancy. Foetal weight was described as large for gestational age (LGA), or small for gestational age (SGA) according to the Foetal Medicine Foundation criteria [30]. All the anthropometric measurements included in the study were performed by {Pone person trained in the EU project “Vitamin D and lifestyle intervention in the prevention of gestational diabetes (GDM) (in Poland)”; Project ID: 242,187.

Every 2–3 weeks, all patients were scheduled for follow–up appointments at the outpatient clinic. At each visit, the authors of the study re–educated the patients regarding the recommended diet and proper treatment, based on the data obtained from the patients’ glucometers, as well as those collected at every appointment.

If confirmed glycaemic levels were above the target concentrations, either the diet was modified, or insulin treatment was initiated.

In the pedometer group, composite outcomes were analysed, including at least 1 of the following complications: gestational hypertension, pre–eclampsia, cholestasis, SGA, LGA, and preterm delivery.

The study was part of the MOCART Study Group registered on clinicaltrials.gov under no NCT04924738, 14/06/2021.

### Blood analysis

Fasting blood was collected at each appointment and immediately transported for analysis. Tina–quant Haemoglobin A1c II test in the Cobas c311 analyser (Roche Diagnostics, Basel, Switzerland) was used to analyse the HbA1c level in the whole blood. The normal range for this test is determined as 4.8–6.0% (29–42mmol/mol) for the non–pregnant population. Triglyceride (TG) and C–reactive protein (CRP) levels were measured with Roche Diagnostics reagents in the Cobas c501 analyser. The reference values for women fluctuate between 0.46–1.71mmol/l. Insulin level was measured during V0 appointment sing enzyme immunoassay (DRG *Insulin ELISA* Kit, DRG Instruments GmbH, Marburg, Germany).

### Statistical analyses

Statistical analyses were performed using the Statistica software, version 13.3 (TIBCO Software, Palo Alto, CA), with installed Medical Bundle, version 4.0.67 (StatSoft Polska Sp. z o.o, Cracow, Poland) and PQStat software, PQStat version 1.8.2.230 (Poznan, Poland). The Shapiro–Wilk test was applied to check the normality of data distribution. Normally distributed variables were assessed using the parametric Student’s t–test, whereas Welch’s t–test was employed for groups with unequal variances. The non–parametric Mann–Whitney U test was applied for non–normally distributed data. Differences between nominal variables were tested using the chi–square test, or the Fisher’s exact test, which was employed when the observed sample size was small (*n* < 5). The authors used Yates’ chi–square test for 3 × 2 contingency tables, and ANOVA with Fisher’s post–hoc tests were applied to compare multiple groups of normally distributed data. In contrast, non–normally distributed data were tested using the non–parametric Kruskal–Wallis ANOVA with a Bonferroni post–hoc test. The authors used the receiver operating characteristic (ROC) curves to establish the analysed variables’ discrimination thresholds P–values were considered statistically significant if *P* < 0.05.

The following sample size calculations were performed. In order to achieve 80% power at a two–sided 5% significance level, the authors planned to include at least 120 (60 per arm) individuals in the study cohort. This, in turn, would allow for detecting a between–group difference in weight gain of 4 kg during pregnancy with a standard deviation of 7 kg in the analysis of differences between the group with pedometers and the control group. The authors assumed a 20% dropout rate.

All patients participating in the study gave written consent to participate. The study was approved by the local bioethics committee of the Poznan University of Medical Sciences.

## Results

Table 1 presents the characteristics of the study group. Notably, the entire group of pregnancies with an average BMI of 37.0 kg/m^2^ (33.0–42.2 kg/m^2^) did not exceed the recommended weight gain, which amounted to 3.0 (–1.0–8.0) kilograms on average. The group achieved the recommended metabolic status represented by the following HbA1C levels in subsequent trimesters: 5.28%, 5.08% and 5.40%, respectively.

**Table 1:** Characteristics of the entire study group

Parameter		*N* = 115
Age [years]		33.1 (5.5)
Gestational age at entry to the study [GW]		16 (12–22)
Body weight before pregnancy [kg]		100 (90–115)
Body weight at the end of pregnancy [kg]		107 (94–119)
Body weight gain during pregnancy [kg]		3.0 (–1.0–8.0)
BMI before pregnancy [kg/cm^2^]		37.0 (33.0–42.2)
BMI at the end of pregnancy [kg/cm^2^]		37.9 (34.0–42.7)
HbA1c V0 [%, mmol/mol]		5.28 (4.92–5.60) 38.97 (36.30–41.33)
HbA1c V1 [%, mmol/mol]		5.08 (4.90–5.48) 37.50 (36.16–40.44)
HbA1c V2 [%, mmol/mol]		5.40 (5.13–5.80) 39.85 (37.86–42.80)

Data presented as Mean (SD) or Median (IQR)

The characteristics of the pedometer group and the control group are presented in Table 2. Significant statistical differences in both groups were found with respect to the patients’ age and history of miscarriage. Despite the lack of statistical significance, a smaller increase in body weight and BMI was observed in patients whose activity was monitored with the use of pedometers. It is of note that family history was positive for obesity, diabetes and hypertension in both groups.

**Table 2:** Characteristics of the analysed groups

Parameter	Control group *N* = 62	Group whit pedometers *N* = 53	*p*
Age [years]	34.2 (5.6)	31.7 (5.1)	0.01*
Gestational age at study entry [GW]	17.1 (5.0)	16.9 (6.0)	0.62 *
Primipara [N; %]	35 (56,5)	23 (43.4)	0.16^
Multipara [N; %]	27 (43.4)	30 (56.6)	0.16^
Patients with a history of miscarriage [N; %]	23 (37)	7 (13)	**< 0.01^**
Body weight before pregnancy [kg]	98 (89–113)	103 (95–118)	0.25*
Weight gain during pregnancy [kg]	4.6 (0.5–8.0)	1.5 (–2.5–6.5)	0.13*
BMI before pregnancy [kg/m^2^]	37.1 (33.1–42.5)	36.8 (33.0–41.1)	0.75*
BMI gain during pregnancy [kg/ m^2^]	1.13 (1.82)	1.07 (1.89)	0.87**
Family history of obesity [N;%]	45 (72)	38 (72)	0.92^
Family history of diabetes [N;%]	36 (58)	37 (70)	0.19^
Family history of HT [N;%]	37 (60)	38 (72)	0.18^

* Mann–Whitney test; ** Student’s t–test; ^ chi^2^ –test;

Following the measurement of skinfolds during appointments in both study groups, it was observed that although BMI did not/did differ between these groups, a significant difference was found between the thickness of the skinfolds at the scapula obtained at V0, which was lower in patients with monitored physical activity.

In the course of pregnancy, a decrease in the delta of all skinfold measurements was found in patients who used pedometers, although it was not significant (Data in supplementary Table 1a).

A comparison of biochemical parameters revealed no significant differences in the percentage of glycated haemoglobin and triglyceride concentrations between the studied groups during the entire pregnancy (Supplement; Table 2a). Both groups presented with an increased level of the HOMA–IR index at the time of inclusion to the study, thus satisfying the criteria of insulin resistance. Supervised physical activity did not significantly affect the concentration of C–reactive protein (CRP). However, it is of note that a decrease in CRP concentration in both groups was observed in the subsequent trimesters, despite the increased insulin resistance during pregnancy (Supplement; Table 2a).

No significant effect of physical activity monitoring was found with regard to the delivery time and the neonate condition. However, the authors observed a crucial impact of controlled activity on macrosomia frequency, which was manifested by its significantly higher frequency in the group of patients who did not use pedometers (Table 3).

**Table 3:** Obstetric outcomes

Parameter	Control group *N* = 62		Group whit pedometers *N* = 53	*p*
Week of completion of delivery [week]	38 (37–38)		38 (37–38)	0.39*
Term births [N; %]	54 (87)		46 (87)	0.96^
Preterm births [N; %]	8 (13)		7 (13)	0.96^
Preterm births 33–36 GW [N; %]	7 (11)		6 (11)	1.00^
Preterm births 28–32 GW [N; %]	1 (1.6)		1 (1.9)	1.00#
Neonate birthweight [kg]	3325 (3065–3680)		3540 (3160–3820)	0.19*
LGA > 90 pc. [N; %]	23 (37)		22 (41)	0.63^
Marcosomia ≥ 4200 g [N,%]	6 (10)		0 (0)	**0.03#**
SGA < 10 pc. [N; %]&	3		1	0.62#
Placental weight [g]	600 (540–760)		633 (575–680)	0.81*
Apgar score 1st minute < 8 [N,%]	6 (10)		3 (6)	0.50#
Apgar score 5th minute < 8 [N,%]	7 (11)		2 (4)	0.17#
Umbilical artery pH	7.30 (7.24–7.33)		7.27 (7.23–7.32)	0.27*

* Mann–Whitney test; ** Student’s t–test; ^ chi^2^–test; # Fisher’s exact test;

& full–term pregnancies after 37 weeks of gestation were included

The group of patients who recorded physical activity was retrospectively divided into two subgroups in terms of the patients’ level of physical activity: individuals whose step counts remained below the guidelines despite supervision, and those who implemented the recommended activity. It is worth bearing in mind that only 45% of patients satisfied the recommended physical activity guidelines (Table 4). The subgroups did not differ with regard to age at the beginning of lifestyle interventions, although a significant difference was observed in the initial body weight and BMI. Both parameters were higher in patients who performed more than 5000 steps daily. Despite higher body weight at the onset of pregnancy, patients who were more active achieved a significantly lower weight gain during pregnancy.

**Table 4:** Characteristics of the subgroups monitoring physical activity with pedometers

Tested parameter	Intervention group < 5000 steps *N* = 29	Intervention group > 5000 steps *N* = 24	*P*	
Age [years]	31.8 (5.1)	31.7 (5.1)	0.93**	
Gestational age at study entry [GW]	18 (6.0)	16 (6.0)	0.19**	
Primipara [N; %]	10 (34.5)	13 (54)	0.15^	
Multipara [N; %]	19 (65.5)	11 (56)	0.15^	
Body weight before pregnancy [kg]	99 (17)	113 (17)	0.005**	
Weight gain during pregnancy [kg]	6.0 (3.0–12.0)	–2.7 (–7.4–0.75)	< 0.001*	
Weight gain from V0 to V1 [kg]	1.0 (–3.0–8.0)	–4.0 (–5.7– − 0.6)	< 0.001*	
BMI before pregnancy [kg/m^2^]	36.0 (4.7)	38.7 (5.3)	0.05**	
BMI gain during pregnancy [kg/ m^2^]	1.40 (0.96–2.31)	0.14 (–1.10–1.2)	< 0.001*	
Family history of obesity [N;%]	18 (62)	15 (62.5)	0.97^	
Family history of diabetes [N;%]	21 (72)	15 (62.5)	0.44^	
Family history of HT [N;%]	2 (7)	5 (21)	0.22#	

* Mann–Whitney test; ** Student’s t–test; ^ chi^2^–test; # Fisher’s exact test;

The analysis of skinfold measurements in both subgroups revealed that at V0 the subgroup, achieving the goal of 5000 steps during pregnancy, demonstrated higher scores in all the evaluated areas. Despite greater thickness of the skinfolds at the beginning of the study, the group of more active patients presented a decrease in the thickness of the skinfolds in each area, and the reduction was significantly lower in the area of ​​the triceps and the scapulae (Supplement; Table 3a).

In turn, biochemical parameters analysis showed no significant differences between the studied subgroups in terms of glycated haemoglobin, triglycerides and CRP (Table 5.). In the subgroup of patients who exceeded 5000 steps per day, only insulin concentrations were significantly higher at the beginning of pregnancy.

**Table 5:** A comparison of biochemical parameters in patients with supervised physical activity

Tested parameter	Intervention group < 5000 steps *N* = 29	Intervention group > 5000 steps *N* = 24	*p*
HbA1c V0 [%, mmol/mol]	5.12 (0.46), 32 (5)	5.33 (0.59), 35 (7)	0.15**
HbA1c V1 [%,mmol/mol]	5.18 (0.43), 33 (5)	5.08 (0.41), 32 (5)	0.39**
HbA1c V2 [%,mmol/mol]	5.47 (0.39), 36 (4)	5.32 (0.52), 35 (6)	0.26**
TG V0 [mg/dl]	169 (59)	170 (58)	0.95**
TG V0 [mg/dl]	222 (164–292)	223 (183–310)	0.80*
TG V0 [mg/dl]	274 (223–391)	288 (230–357)	0.57*
CRP V0 [mg/l]	7.15 (3.24–14.13)	6.85 (5.36–12.20)	0.39*
CRP V1 [mg/l]	7.29 (3.84–12.30)	8.37 (4.59–15.06)	0.62*
CRP V2 [mg/l]	6.79 (3.10–10.68)	6.32 (3.57–8.65)	0.80*
FBG V0 [mg/dl, mmol/l]	102 (96–112), 5.66 (5.33–6.22)	101 (96–105), 5.61 (5.33–5.83)	0.59*
Insulin V0 [uU/ml]	18.03 (12.56–23.84)	36.38 (16.35–48.50)	0.048*
HOMA IR V0	4.29 (2.76–6.19)	9.04 (4.14–13.73)	0.09*

* Mann–Whitney test; ** Student’s t–test;

The perinatal outcome in the group of patients performing more than 5000 steps did not demonstrate significant differences compared to the less active group (Table 6). However, in children of patients leading an active lifestyle, a lower rate of both hypoglycaemic and hyperbilirubinemia events was demonstrated. Moreover, no cases of congenital anomalies or respiratory distress syndrome were found in either group.

**Table 6:** Perinatal outcome in patients in relation to physical activity

Tested parameter	Intervention group < 5000 steps *N* = 29	Intervention group > 5000 steps *N* = 24	*p*	
Week of completion of delivery [week]	38 (37–38)	37 (37–38)	0.22*	
Term births [N; %]	26 (90)	20 (83)	0.69#	
Preterm births [N; %]	3 (10)	4 (17)	0.69#	
Preterm births 33–36 GW [N; %]	3 (10)	3 (10)	1.00#	
Preterm births 28–32 GW [N; %]	0 (0.0)	1 (4)	0.45#	
Neonate birthweight [kg]	3560 (3280–3880)	3425 (2963–3740)	0.28*	
LGA > 90 pc. [N; %]	13 (45)	9 (37.5)	0.59^	
SGA < 10 pc. [N; %]&	0 (0)	1 (4)	0.45#	
Placental weight [g]	649 (109)	611 (130)	0.27**	
Apgar score 1st minute < 8 [N,%]	2 (7)	1 (4)	1.00#	
Apgar score 5th minute < 8 [N,%]	2 (7)	0 (0)	0.49#	
Umbilical artery pH	7.27 (7.22–7.33)	7.28 (7.23–7.31)	0.77*	
Hyperbilirubinemia	16 (55)	9 (37.5)	0.20^	
Hypoglycaemia	4 (14)	1 (4)	0.36#	

* Mann–Whitney test; ** Student’s t–test; ^ chi^2^–test; # Fisher’s exact test;

& full–term pregnancies after 37 weeks of gestation were included

A pooled analysis indicated that the patients without pedometers were significantly older than both subgroups with pedometers. It was clearly demonstrated that although the patients from the intervention subgroup with over 5000 steps were characterized by a higher pre-pregnancy body weight and BMI, they showed significant differences in weight gain during pregnancy and in weight gain between V0 and V1 appointments, compared to the control group and the subgroup under 5000 steps. This association is presented in Supplement; Table 4a.

The subgroup of patients with pedometer–supervised physical activity was divided according to the complications observed in pregnant women and their offspring: gestational hypertension, pre–eclampsia, cholestasis, SGA, LGA, preterm delivery (poor composite outcome). Among patients without poor composite outcome, a significant difference was demonstrated in body weight before pregnancy and in each trimester and in their BMIs, which were significantly lower. Additionally, both the neonate and placental weight was significantly lower in this group (Table 7).

**Table 7:** The analysis of the pedometer–supervised group – a comparison of the group with a composite outcome of obstetric complications and the group without complications

Tested parameter	Poor composite outcome		*P*
	NO *N* = 21	YES *N* = 32	
Age [years]	32.3 (5.1)	31.4 (5.1)	0.93**
Gestational age at study entry [GW]	17 (7)	17 (6)	0.69**
Body weight before pregnancy [kg]	98 (18)	110 (15)	0.02**
Body weight V0 [kg]	97 (15)	110 (15)	0.003**
Body weight V1 [kg]	97 (15)	112 (14)	0.001**
Body weight V2 [kg]	100 (15)	114 (15)	0.001**
BMI V0 [kg/m^2^]	34.5 (4.5)	39.0 (4.8)	0.001**
BMI V1 [kg/m^2^]	34.6 (4.6)	39.5 (4.6)	< 0.001**
BMI V2 [kg/m^2^]	35.4 (4.2)	40.2 (4.9)	< 0.001**
Neonate birthweight [kg]	3340 (3045–3520)	3740 (3350–3905)	0.002*
Placental weight [g]	584 (75)	663 (130)	**< 0.01$**

* Mann–Whitney test ** Student’s t–test $ Welch’s t–test

**Fig. 3:**
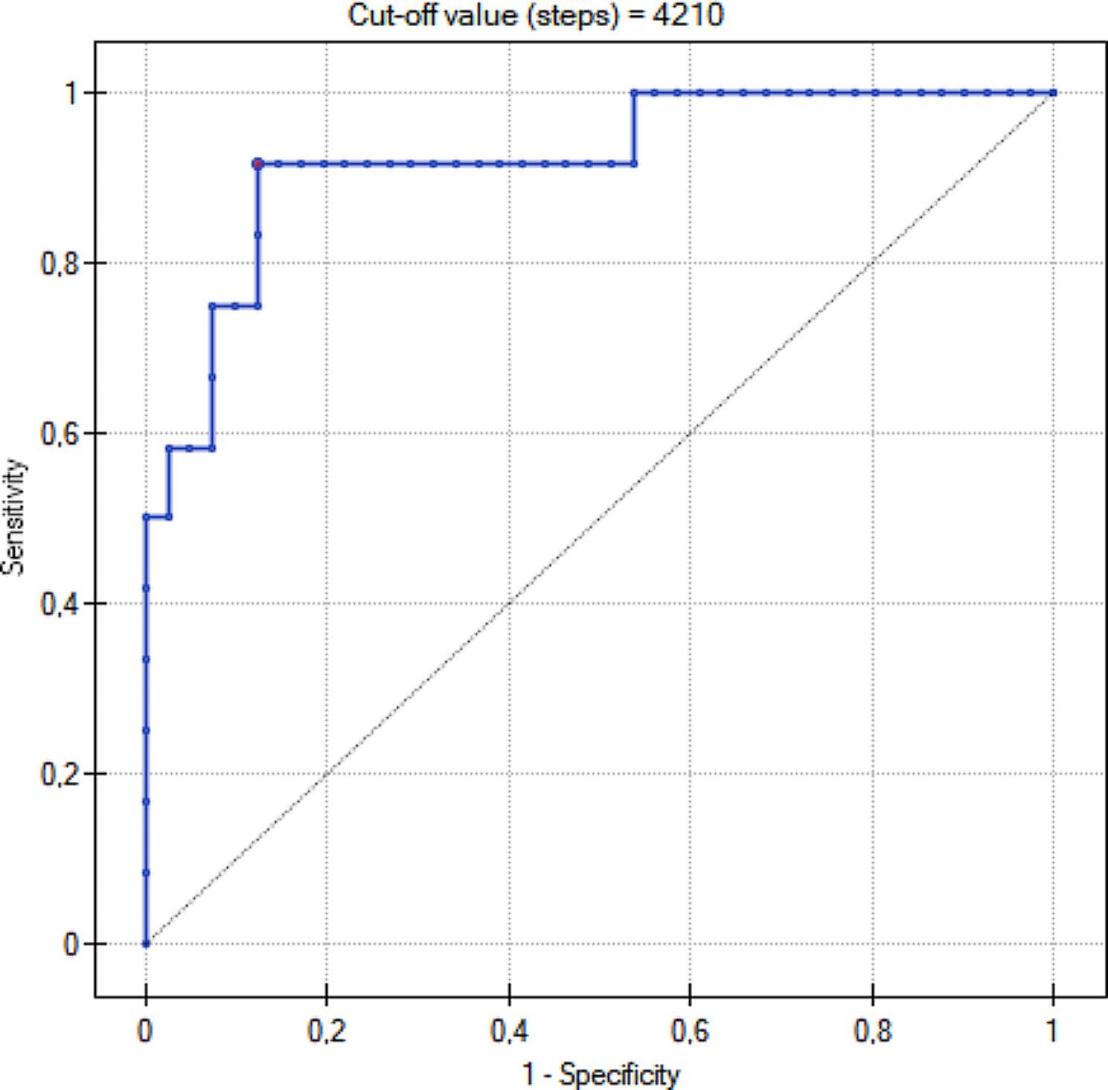
The association between the recorded physical activity (the mean number of steps per day) and the risk of abnormal gestational weight gain (gestational weight gain > 7 kg) – the ROC analysis

Due to a relatively small number of complications that occurred in the group using pedometers, it was possible to model the ROC curves only for LGA, hyperbilirubinemia, negative perinatal composite outcome and weight gain above the recommended level at the beginning of pregnancy (Fig. 3.). No statistical significance was shown for the first three variables. However, the obtained ROC curve for weight gain above the guidelines indicated a statistically significant cut–off point for this group at the level of 4210 steps/day (p–0.00001). Data are presented in Table 8.

**Table 8:** The mean number of steps and the risk of perinatal complications – the ROC analysis

Tested parameter	AUC	Cut–off value (steps)	Sensitivity	Specificity	*p*–value
LGA	0.57	5433	0.91	0.39	0.36
Hyperbilirubinemia	0.53	4340	0.39	0.72	0.67
Negative perinatal composite outcome	0.55	5833	0.88	0.33	0.52
Gestational weight gain > 7 kg	0.92	4210	0.92	0.88	0.00001

Negative perinatal composite outcome: gestational hypertension, pre–eclampsia, cholestasis, SGA, LGA, preterm delivery

## Discussion

Physical activity is a widely recognised method to improve the quality of life and reduce factors negatively affecting health, mainly aiming to reduce the complications of metabolic diseases, obesity and diabetes [31]. Our study attempted to evaluate the impact of physical activity on the metabolic status of pregnant women with early gestational diabetes, as well as on obesity and neonatal outcomes. Previous studies have demonstrated that exercise during pregnancy decreases the risk of gestational diabetes and reduces weight gain during pregnancy [18, 32, 33]. Moreover, various types of activities have also been the subject of research with regard to their impact on metabolic control in pregnant women suffering from gestational diabetes. Our research focused on walking and the step count. Coe et al. found that a half-hour walk may reduce glucose concentrations for up to two hours after the activity and provide better glucose control than that displayed by pregnant women leading a sedentary lifestyle [34]. In our study, the authors instructed the patients with regard to the suggested level of physical activity and recommended taking a minimum of 5000 steps daily. Nevertheless, only 45% of the monitored group performed the recommended number of steps. According to the Indian Wings study, only 16.8% of patients achieved the recommended level of physical activity, although this percentage increased to 26.5% following the intervention. It was also observed that pregnant women with GDM led a sedentary lifestyle more frequently than healthy pregnant women [35]. Therefore, our results appear promising in comparison to these data.

It is of note that not all guidelines regarding physical activity during pregnancy precisely indicate the recommended number of steps during the day. The standards of the Polish Society of Gynecologists and Obstetricians in managing women with diabetes recommend 150 min of physical activity per week, divided into 30–minute sessions [26]. However, they do not refer to the number of the recommended steps per day. Our study determined a limit of 5000 steps as the recommended daily physical activity. In fact, according to the conducted research, activity under 5000 steps daily is considered a sedentary lifestyle [28]. The study of Hayashi demonstrated that 6000 steps a day allowed for the maintenance of lower glycaemic concentrations in patients with gestational diabetes than in the group performing fewer steps [36]. The analysis of the ROC curves in our study indicated that activity at the level of 4210 steps per day resulted in achieving the expected weight gain goal during pregnancy in women with obesity. Furthermore, the majority of patients who were physically active at this level gained less than 7 kg during pregnancy.

Patients with obesity and gestational diabetes participating in our study achieved outstanding results in terms of metabolic parameters. The mean glycated haemoglobin levels in the group were within the recommended ranges. Similarly, the average weight gain during pregnancy among pregnant women from the study group did not exceed the recommended level [26]. Complex education regarding optimal diet and physical activity promotion among the studied group contributed to obtaining favourable results. This, in turn, is in line with the results published by the DALI group, which showed that promoting healthy eating and physical activity was the preferred strategy for limiting gestational weight gain [37]. Proper metabolic compensation was also reflected in positive neonatal outcomes. In the studied group, only six newborns had a birth weight of more than 4200 g, which was defined as macrosomia. In the subgroup of patients who performed more than 5000 steps per day (High–PE), a significantly smaller weight gain was observed from V0 to V1 and during the entire pregnancy compared to patients in the subgroup with fewer steps (Low–PE). Additionally, a total reduction in body weight during pregnancy was observed in the High- PE subgroup of patients. Researchers from the Atkinson’s group set a similar aim for their research. The average number of steps taken in their study group amounted to 7043 at the assumed 10,000 steps. In the group of patients with obesity, despite taking more than 7000 steps a day, the recommended weight gain was not achieved [38]. Our study proved that adhering to dietary recommendations with appropriate physical activity allowed the patients to achieve the recommended weight gain and even weight reduction during pregnancy, which did not adversely affect foetal development and neonatal outcomes.

The study group displayed a high rate of caesarean section, amounting to 63%. The high number of C-section procedures in overweight and gendered patients corresponds to the increased risk of C-section due to a previously performed caesarean section, difficult/non–progressive delivery, failed labour induction, and foetal distress in pregnant women with overweight and obesity [39]. The number of surgical deliveries conducted due to intrapartum indications was only 14%, which represented a smaller proportion than that obtained by Athukoral et al., who reported 24.9% and 29.8% in patients with overweight and obesity, respectively. It is interesting to note that, in the same study, a 16.9% increase in the number of caesarean sections was observed in comparison to women with normal and elevated BMI [40]. Additionally, our research analysed the number of preterm births in the study group, and the authors found no differences between the studied groups in gestational weeks and the percentage of preterm births. Nevertheless, an increase in preterm birth rates was recorded, i.e. in the control and pedometer–monitored groups by 13%, in the < 5000 steps subgroup by 10%, and in the > 5000 steps subgroup by 17%. The overall increase in preterm births in our groups was higher than the results obtained by Sun et al. (6.0% in patients with overweight, 6.8% in individuals with obesity). However, the pregnancies of all patients in our study comprised pregnant women with early GDM [41]. The majority of preterm births in the group of pregnant women in the presented study occurred between 33rd and 36th + 6 weeks of gestation. Only two deliveries between 28th and 32nd + 6 weeks were reported, hence, it may be concluded that the recommended physical activity did not affect the occurrence of a preterm delivery.

Although the results of other conducted studies are inconsistent, they indicate that mothers with pre-pregnancy diabetes show abnormal pro–inflammatory protein/cytokine concentrations, such as IL–8, IL–6, CRP, TNF–α and IFN–γ. Studies suggest that the presence of inflammation may affect the growing foetus by limiting various circulatory functions [42]. In our studied group, an increase in C–reactive protein (CRP) levels throughout pregnancy was not observed, and the supervised physical activity did not trigger inflammatory processes.

Various authors emphasise that maternal obesity is associated with excessive neonatal obesity, which constitutes a risk factor for developing obesity and type 2 diabetes (T2DM) in childhood and adulthood [43, 44]. Additionally, the placenta may mediate adverse effects of maternal obesity on both foetal development and neonatal obesity. Anika et al. showed that elevated maternal insulin and placental insulin receptor abundance affected placental lipid metabolism, even in normoglycaemic conditions. The authors of the presented study measured the fasting insulin concentration at the first appointment at the beginning of pregnancy. As a result, significantly increased fasting insulin levels were found in those patients who subsequently achieved their physical activity goals. Thus, it is possible to hypothesize that they achieved outstanding perinatal results due to increased physical activity despite presenting hyperinsulinemia at the beginning of pregnancy.

Although fatty acids are an efficient substrate for triglycerides (TG) synthesis and storage in foetal adipose tissue [45, 46], according to the results observed in our study, triglyceride levels did not differ significantly between the subgroups.

In our group, the percentage of LGA newborns amounted to 39%. Notably, the subgroup performing more than 5000 steps per day presented a higher body weight and BMI at V0, as well as showed higher insulin concentrations and HOMA–IR index values. In spite of poorer baseline parameters, the authors did not find a case of macrosomia in this group. Interestingly, all macrosomic newborns were delivered in the group of patients without pedometers, which constituted a significant differentiating factor between the groups. According to our observations, activity exceeding 5000 steps did not result in a higher percentage of SGA newborns, which is similar to the results obtained by Neal et al. [47]. Although obesity is generally recognised as a risk factor for SGA, our study showed that obesity affected LGA, yet had no significant effect on the incidence of SGA.

A combined abnormal obstetric outcome analysis confirmed that a higher body weight and BMI in the course of pregnancy resulted in a statistically significantly higher incidence of such abnormal outcomes.

Pre-pregnancy symptoms of overweight and obesity constitute a more and more frequent challenge for therapeutic teams who face the need to manage pregnancies, presenting with high–risks both for the mother and for the child. Postponing conception until the recommended maternal BMI is achieved was shown to improve obstetric and neonatal outcomes [48]. However, if pregnancy occurs in a patient with overweight or obesity, a balanced diet and regular physical activity may contribute to achieving a positive obstetric outcome.

## Conclusions

Monitoring the activity of pregnant patients with gestational diabetes and obesity by pedometers did not have a significant impact on their metabolic control and weight gain. Supervision of pregnant women helps to reduce the number of macrosomic newborns. Furthermore, physical activity positively affects weight loss in pregnant women with obesity diseases, as well as contributes to improved obstetric and neonatal outcomes. A very valuable result is the analysis which shows that taking less than 4210 steps a day promotes weight gain during pregnancy of more than 7 kg. Therefore, taking more steps than this cut-off point should help maintain the recommended weight gain.

The strength of our study is analysing the impact of physical activity on patients’ health, the development of pregnancy and on the condition of newborns. It may serve as the basis for establishing recommendations for pregnant women regarding the suggested number of walking steps. In contrast, the limitation of the study are the small size of the study group and the lack of a control group in which the patients would not perform any physical activity at all. However, establishing such a control group would be unethical, due to the strong evidence for the beneficial effects that physical activity exerts on human health. Another limitation of the study was the lack of clear criteria for the diagnosis of GDM in the first half of pregnancy.

### Electronic supplementary material

Below is the link to the electronic supplementary material.

Supplementary Material 1

## Data Availability

The authors make it possible to share the data and materials used to develop and interpret the results. Please contact Lukasz Adamczak- corresponding author.

## List of abbreviations

BMI: Body mass index
CRP: C-reactive protein
EU: European Union
FBG: Fasting banding glucose
GDM: Gestational diabetes mellitus
GW: Gestational week
GWG: Gestational weight gain
HbA_1C−_: Glycated hemoglobin
HOMA-IR: Homeostatic Model Assessment for Insulin Resistance
HT: Hypertension
IADPSG: International Association Diabetes Pregnancy Study Group
IFN–γ: Interferon γ
IL-6: Interleukin 6
IL-8: Interleukin 8
IOM: Institute of Medicine
LGA: Large for gestational age
NP: Non-pedometers study group
OGTT: Oral glucose tolerance test
P: Pedometers study group
ROC: Receiver operating characteristic
SGA: Small for gestational agr
T2DM: Type 2 diabetes
TG: Triglyceride
TNF–α: Tumour necrosis factor α
WHO: World Health Organisation

## References

[1] Chu SY, Callaghan WM, Kim SY, Schmid CH, Lau J, England LJ et al. Maternal obesity and risk of gestational diabetes mellitus. Diabetes Care. 2007;30. 10.2337/dc06-2559a 17416786

[2] Pirkola J, Pouta A, Bloigu A, Hartikainen AL, Laitinen J, Järvelin MR et al. Risks of overweight and abdominal obesity at age 16 years associated with prenatal exposures to maternal prepregnancy overweight and gestational diabetes mellitus. Diabetes Care. 2010;33. 10.2337/dc09-1871 PMC2858187 20427685

[3] World Health Organization (WHO). Obesity and Overweight. Available online: https://www.who.int/news–room/fact–sheets/detail/obesity–and–overweight (accessed on 20 January 2023).

[4] Blüher M. Obesity: global epidemiology and pathogenesis. Nat Reviews Endocrinol. 2019;15. 10.1038/s41574-019-0176-8 30814686

[5] Zehravi M, Maqbool M, Ara I. Correlation between obesity, gestational diabetes mellitus, and pregnancy outcomes: an overview. Int J Adolesc Med Health. 2021;33. 10.1515/ijamh-2021-0058 34142511

[6] Riley L, Wertz M, McDowell I. Obesity in pregnancy: risks and management. Am Family Phys. 2018;97. 29763261

[7] Jakicic JM, Davis KK. Obesity and physical activity. Psychiatr Clin North Am. 2011;34. 10.1016/j.psc.2011.08.009 22098807

[8] Martínez–Vizcaíno V, Álvarez–Bueno C. Cavero–Redondo I. Diet in the management of weight loss. Nutrients. 2021;13. 10.3390/nu13041306 PMC8071278 33920924

[9] Gila-Díaz A, Witte Castro A, Herranz Carrillo G, Singh P, Yakah W, Arribas SM, Ramiro-Cortijo D. Assessment of adherence to the healthy food pyramid in pregnant and lactating women. Nutrients. 2021;13. 10.3390/nu13072372 PMC8308771 34371882

[10] Oken E, Kleinman KP, Belfort MB, Hammitt JK, Gillman MW. Associations of gestational weight gain with short– and longer–term maternal and child health outcomes. Am J Epidemiol. 2009;170. 10.1093/aje/kwp101 PMC2727269 19439579

[11] Streuling I, Beyerlein A, Von Kries R. Can gestational weight gain be modified by increasing physical activity and diet counseling? A meta–analysis of interventional trials. Am J Clin Nutr. 2010;92. 10.3945/ajcn.2010.29363 20668049

[12] Chin JR, Krause KM, Østbye T, Chowdhury N, Lovelady CA, Swamy GK. Gestational weight gain in consecutive pregnancies. Am J Obstet Gynecol. 2010;203. 10.1016/j.ajog.2010.06.038 20816151

[13] Rasmussen KM, Yaktine AL, Institute of Medicine (U.S.) and National Research Council (U.S (2009). Committee to reexamine IOM Pregnancy Weight Guidelines. Weight gain during pregnancy: reexamining the guidelines.

[14] Dalfra’ MG, Burlina S, Lapolla A. Weight gain during pregnancy: a narrative review on the recent evidences. Diabetes Res Clin Pract. 2022;188. 10.1016/j.diabres.2022.109913 35568262

[15] Santos S, Voerman E, Amiano P, Barros H, Beilin LJ, Bergström A et al. Impact of maternal body mass index and gestational weight gain on pregnancy complications: an individual participant data meta–analysis of European, north American and Australian cohorts. BJOG. 2019;126. 10.1111/1471-0528.15661 PMC6554069 30786138

[16] Voerman E, Santos S, Inskip H, Amiano P, Barros H, Charles MA et al. Association of Gestational Weight Gain with adverse maternal and infant outcomes. JAMA. 2019;321. 10.1001/jama.2019.3820 PMC6506886 31063572

[17] Champion ML, Harper LM. Gestational weight gain: update on outcomes and interventions. Curr Diab Rep. 2020;20. 10.1007/s11892-020-1296-1 32108283

[18] Barakat R, Refoyo I, Coteron J, Franco E. Exercise during pregnancy has a preventative effect on excessive maternal weight gain and gestational diabetes. A randomised controlled trial. Braz J Phys Ther. 2019;23. 10.1016/j.bjpt.2018.11.005 PMC6428908 30470666

[19] Teede HJ, Bailey C, Moran LJ, Bahri Khomami M, Enticott J, Ranasinha S et al. Association of antenatal diet and physical activity–based interventions with gestational weight gain and pregnancy outcomes: a systematic review and meta–analysis. JAMA Intern Med. 2022;182. 10.1001/jamainternmed.2021.6373 PMC8689430 34928300

[20] Sandborg J, Söderström E, Henriksson P, Bendtsen M, Henström M, Leppänen MH et al. Effectiveness of a smartphone app to promote healthy weight gain, diet, and physical activity during pregnancy (healthymoms): randomised controlled trial. JMIR Mhealth Uhealth. 2021;9. 10.2196/26091 PMC7995071 33704075

[21] Yew TW, Chi C, Chan SY, van Dam RM, Whitton C, Lim CS et al. A randomised controlled trial to evaluate the effects of a smartphone application–based lifestyle coaching program on gestational weight gain, glycemic control, and maternal and neonatal outcomes in women with gestational diabetes mellitus: the smart–gdm study. Diabetes Care. 2021;44. 10.2337/dc20-1216 PMC7818327 33184151

[22] McCurdy RJ, Delgado DJ, Baxter JK, Berghella V. Influence of weight gain on risk for cesarean delivery in obese pregnant women by class of obesity: pregnancy risk assessment monitoring system (PRAMS). J Maternal–Fetal Neonatal Med. 2022;35. 10.1080/14767058.2020.1802714 32762274

[23] Langer O, Yogev Y, Most O, Xenakis EMJ. Gestational diabetes: the consequences of not treating. In: Am J Obstet Gynecol. 2005;192. 10.1016/j.ajog.2004.11.039 15846171

[24] Langer O, Yogev Y, Xenakis EMJ, Brustman L. Overweight and obese in gestational diabetes: the impact on pregnancy outcome. Am J Obstet Gynecol. 2005;192. 10.1016/j.ajog.2004.12.049 15970805

[25] Garvey WT, Mechanick JI, Brett EM, Garber AJ, Hurley DL, Jastreboff AM et al. American association of clinical endocrinologists and American college of endocrinology comprehensive clinical practice guidelines for medical care of patients with obesity. Endocr Pract. 2016;22. 10.4158/EP161365.GL 27219496

[26] Wender–Ozegowska E, Bomba–Opoń D, Brazert J, Celewicz Z, Czajkowski K, Gutaj P et al. Standards of Polish society of gynecologists and obstetricians in management of women with diabetes. Ginekol Pol. 2018;89. 10.5603/GP.a2018.0059 30010185

[27] Diagnostic criteria. And classification of hyperglycaemia first detected in pregnancy: a World Health Organization guideline. Diabetes Res Clin Pract. 2014;103. 10.1016/j.diabres.2013.10.012 24847517

[28] Tudor-Locke C, Bassett DR Jr. How many steps/day are enough? Preliminary pedometer indices for public health. Sports Med. 2004;34. 10.2165/00007256-200434010-00001 14715035

[29] Caughey AB, Turrentine M. ACOG PRACTICE BULLETIN: gestational diabetes mellitus. Obstet Gynecol. 2018;131. 10.1097/AOG.0000000000002501 29370047

[30] Nicolaides KH, Wright D, Syngelaki A, Wright A, Akolekar R. Fetal medicine foundation fetal and neonatal population weight charts. Ultrasound Obstet Gynecol. 2018;52. 10.1002/uog.19073 29696704

[31] Di Biase N, Balducci S, Lencioni C, Bertolotto A, Tumminia A, Dodesini AR et al. Review of general suggestions on physical activity to prevent and treat gestational and pre–existing diabetes during pregnancy and in postpartum. Nutr Metabolism Cardiovasc Dis. 2019;29. 10.1016/j.numecd.2018.10.013 30642790

[32] Wang C, Wei Y, Zhang X, Zhang Y, Xu Q, Sun Y et al. A randomised clinical trial of exercise during pregnancy to prevent gestational diabetes mellitus and improve pregnancy outcome in overweight and obese pregnant women. Am J Obstet Gynecol. 2017;216. 10.1016/j.ajog.2017.05.009 28502755

[33] Davenport MH, Ruchat SM, Poitras VJ, Jaramillo Garcia A, Gray CE, Barrowman N et al. Prenatal exercise for the prevention of gestational diabetes mellitus and hypertensive disorders of pregnancy: a systematic review and meta–analysis. Br J Sports Med. 2018;52. 10.1136/bjsports-2018-099355 30337463

[34] Coe DP, Conger SA, Kendrick JM, Howard BC, Thompson DL, Bassett DR et al. Postprandial walking reduces glucose levels in women with gestational diabetes mellitus. Applied Physiology, Nutrition and Metabolism. 2018;43. 10.1139/apnm-2017-0494 29272606

[35] Anjana RM, Sudha V, Lakshmipriya N, Anitha C, Unnikrishnan R, Bhavadharini B et al. Physical activity patterns and gestational diabetes outcomes – the wings project. Diabetes Res Clin Pract. 2016;116. 10.1016/j.diabres.2016.04.041 27321343

[36] Hayashi A, Oguchi H, Kozawa Y, Ban Y, Shinoda J, Suganuma N. Daily walking is effective for the management of pregnant women with gestational diabetes mellitus. J Obstet Gynecol Res. 2018;44. 10.1111/jog.13698 PMC6174974 29974564

[37] Broekhuizen K, Simmons D, Devlieger R, van Assche A, Jans G, Galjaard S et al. Cost–effectiveness of healthy eating and/or physical activity promotion in pregnant women at increased risk of gestational diabetes mellitus: economic evaluation alongside the DALI study, a European multicenter randomised controlled trial. Int J Behav Nutr Phys Activity. 2018;15. 10.1186/s12966-018-0643-y PMC5853142 29540227

[38] Atkinson SA, Maran A, Dempsey K, Perreault M, Vanniyasingam T, Phillips SM et al. Be healthy in pregnancy (BHIP): a randomized controlled trial of nutrition and exercise intervention from early pregnancy to achieve recommended gestational weight gain. Nutrients. 2022;14. 10.3390/nu14040810 PMC8879855 35215461

[39] Fernández Alba JJ, Paublete Herrera C, Vilar Sanchez A, Gonzalez–Macias C, Castillo Lara M, Torrejón R et al. Indications of caesarean section in overweight and obese versus normal–weight pregnant women: a retrospective cohort study. J Maternal–Fetal Neonatal Med. 2018;31. 10.1080/14767058.2017.1285894 28118780

[40] Athukorala C, Rumbold AR, Willson KJ, Crowther CA. The risk of adverse pregnancy outcomes in women who are overweight or obese. BMC Pregnancy Childbirth. 2010;10. 10.1186/1471-2393-10-56 PMC2949787 20849609

[41] Sun Y, Shen Z, Zhan Y, Wang Y, Ma S, Zhang S et al. Effects of prepregnancy body mass index and gestational weight gain on maternal and infant complications. BMC Pregnancy Childbirth. 2020;20. 10.1186/s12884-020-03071-y PMC7336408 32631269

[42] Denizli M, Capitano ML, Kua KL. Maternal obesity and the impact of associated early–life inflammation on long–term health of offspring. Front Cell Infect Microbiol. 2022;12. 10.3389/fcimb.2022.940937 PMC9523142 36189369

[43] Schack–Nielsen L, Michaelsen KF, Gamborg M, Mortensen EL, Sørensen TIA. Gestational weight gain in relation to offspring body mass index and obesity from infancy through adulthood. Int J Obes. 2010;34. 10.1038/ijo.2009.206 19918246

[44] Gaillard R, Santos S, Duijts L, Felix JF. Childhood health consequences of maternal obesity during pregnancy: a narrative review. Ann Nutr Metab. 2017;69. 10.1159/000453077 27855382

[45] Anam AK, Cooke KM, Dratver MB, O’Bryan JV, Perley LE, Guller SM et al. Insulin increases placental triglyceride as a potential mechanism for fetal adiposity in maternal obesity. Mol Metab. 2022;64. 10.1016/j.molmet.2022.101574 PMC9440306 35970449

[46] Harreiter J, Mendoza LC, Simmons D, Desoye G, Devlieger R, Galjaard S et al. Vitamin D3 supplementation in overweight/obese pregnant women: no effects on the maternal or fetal lipid profile and body fat distribution—a secondary analysis of the multicentric, randomized, controlled vitamin d and lifestyle for gestational diabetes prevention trial (DALI). Nutrients. 2022;14. 10.3390/nu14183781 PMC9503968 36145157

[47] Neal K, Ullah S, Glastras SJ. Obesity class impacts adverse maternal and neonatal outcomes independent of diabetes. Front Endocrinol (Lausanne). 2022;13. 10.3389/fendo.2022.832678 PMC8987983 35399939

[48] Mariona FG. Perspectives in obesity and pregnancy. Women’s Health. 2016;12. 10.1177/1745505716686101 PMC5373261 29334009

