# Supplementary material for: Physical activity, gestational weight gain in obese patients with early gestational diabetes and the perinatal outcome – a randomised–controlled trial
Source: BMC Pregnancy Childbirth. 2024 Feb 2;24:104. doi: 10.1186/s12884-024-06296-3 (PMC10836025; doi:10.1186/s12884-024-06296-3)
Supplement: Supplementary file 1 — Supplementary Material 1 [file 12884_2024_6296_MOESM1_ESM.docx]

| Table 1a. A comparison of anthropometric measurements. | | | |  |
| --- | --- | --- | --- | --- |
| Parameter | Control group  N = 62 | Group whit pedometers  N = 53 | p | |
| Neck circumference V0 [cm] | 37.1 (2.3) | 37.3 (2.4) | 0.62** | |
| Neck circumference V1 [cm] | 37.3 (2.2) | 37.4 (2.2) | 0.75** | |
| Neck circumference V2 [cm] | 37.5 (2.2) | 37.6 (2.2) | 0.83** | |
| Delta neck circumference V0–V2 [cm] | 0.3 (–0.4 – 1.2) | 0 (–0.5 – 1.0) | 0.46* | |
| Triceps fold V0 [cm] | 2.97 (2.66 – 3.41) | 2.69 (2.35 – 3.30) | 0.09* | |
| Triceps fold V1 [cm] | 3.01 (0.63) | 2.80 (0.76) | 0.12** | |
| Triceps fold V2 [cm] | 2.90 (2.37 – 3.48) | 2.90 (2.28 – 3.34) | 0.38* | |
| Delta Triceps fold V0–V2 [cm] | –0.11 (–0.50 – 0.28) | –0.08 (–0.30 – 0.25) | 0.61* | |
| Biceps fold V0 [cm] | 2.00 (1.58 – 2.36) | 1.90 (1.38 – 2.93) | 0.89* | |
| Biceps fold V1 [cm] | 2.08 (1.76 – 2.54) | 2.03 (1.47 – 3.00) | 0.78* | |
| Biceps fold V2 [cm] | 2.14 (1.82 – 2.70) | 2.05 (1.36 – 2.80) | 0.26* | |
| Delta Biceps fold V0–V2 [cm] | 0.12 (–0.14 – 0.56) | –0.03 (–0.28 – 0.30) | 0.05* | |
| Scapula fold V0 [cm] | 3.47 (0.64) | 3.16 (0.78) | 0.03** | |
| Scapula fold V1 [cm] | 3.47 (0.69) | 3.23 (0.82) | 0.11** | |
| Scapula fold V2 [cm] | 3.44 (0.78) | 3.27 (0.87) | 0.29** | |
| Delta Scapula fold V0–V2 [cm] | 0.05 (–0.34 – 0.36) | –0.06 (–0.32 – 0.39) | 0.99* | |
| Hip fold V0 [cm] | 2.92 (2.46 – 3.44) | 2.76 (2.34 – 3.34) | 0.24* | |
| Hip fold V1 [cm] | 2.88 (2.44 – 3.18) | 2.75 (2.24 – 3.16) | 0.52* | |
| Hip fold [cm] | 2.96 (2.40 – 3.64) | 2.92 (2.30 – 3.46) | 0.63* | |
| Delta Hip fold [cm] | –0.06 (–0.44 – 0.40) | –0.15 (–0.28 – 0.24) | 0.96* | |

* Mann–Whitney test; ** Student’s t–test

| \| Table 2a. A comparison of biochemical parameters. \| \| \|  \|  \| \|  \| \| --- \| --- \| --- \| --- \| --- \| --- \| --- \| \| Tested parameter \| Control group  N = 62 \| Group with pedometers  N = 53 \| \| \| P \| \| \| \| HbA1c V0 [%, mmol/mol] \| 5.35 (4.99 – 5.60) , 35 (31–38) \| 5.14 (4.89 – 5.60), 33 (30–38) \| \| \| 0.21* \| \| \| \| HbA1c V1 [%, mmol/mol] \| 5.10 (4.90 – 5.51), 32 (30–37) \| 5.08 (4.87 – 5.42), 32 (30–36) \| \| \| 0.62* \| \| \| \| HbA1c V2 [%, mmol/mol] \| 5.40 (5.13 – 5.86), 36 (33–41) \| 5.38 (5.12 – 5.79), 35 (32–40) \| \| \| 0.76* \| \| \| \| TG V0 [mg/dl] \| 169 (123 – 210) \| 165 (130 – 205) \| \| \| 1.00* \| \| \| \| TG V1 [mg/dl] \| 216 (170 – 278) \| 222 (167 – 305) \| \| \| 0.75* \| \| \| \| TG V2 [mg/dl] \| 299 (237 – 385) \| 276 (230 – 389) \| \| \| 0.52* \| \| \| \| CRP V0 [mg/l] \| 8.57 (5.26–14.04) \| 7.09 (4.82–13.33) \| \| \| 0.17* \| \| \| \| CRP V1 [mg/l] \| 6.91 (4.55–10.44) \| 7.48 (3.96–13.86) \| \| \| 0.62* \| \| \| \| CRP V2 [mg/l] \| 5.86 (3.64–10.47) \| 6.60 (3.45–9.20) \| \| \| 0.73* \| \| \| \| FBG V0 [mg/dl] \| 102 (98–110) \| 101 (96–107) \| \| \| 0.96* \| \| \| \| Insulin V0 [uU/ml] \| 15.53 (11.50–28.27) \| 20.27 (13.15–38.94) \| \| \| 0.96* \| \| \| \| HOMA IR V0 \| 4.15 (2.73–6.98) \| 5.44 (3.25–9.84) \| \| \| 0.15* \| \| \|   *Mann–Whitney test  Table 3a. A comparison of anthropometric measurements in patients performing supervised physical activity. | | | |  |
| --- | --- | --- | --- | --- | --- | --- | --- | --- | --- | --- | --- | --- | --- | --- | --- | --- | --- | --- | --- | --- | --- | --- | --- | --- | --- | --- | --- | --- | --- | --- | --- | --- | --- | --- | --- | --- | --- | --- | --- | --- | --- | --- | --- | --- | --- | --- | --- | --- | --- | --- | --- | --- | --- | --- | --- | --- | --- | --- | --- | --- | --- | --- | --- | --- | --- | --- | --- | --- | --- | --- | --- | --- | --- | --- | --- | --- | --- | --- | --- | --- | --- | --- | --- | --- | --- | --- | --- | --- | --- | --- | --- | --- | --- | --- | --- | --- | --- | --- | --- | --- | --- | --- | --- | --- | --- | --- | --- | --- | --- | --- | --- | --- | --- | --- | --- |
| Tested parameter | Intervention group <5000 steps  N = 29 | Intervention group >5000 steps  N = 24 | p | |
| Neck circumference V0 [cm] | 37.1 (2.2) | 37.7 (2.7) | 0.34** | |
| Neck circumference V1 [cm] | 37.2 (1.8) | 37.7 (2.6) | 0.35** | |
| Neck circumference V2 [cm] | 37.6 (1.9) | 37.5 (2.7) | 0.88** | |
| Delta neck circumference [cm] | 0.8 (–0.6 – 1.1) | 0.0 (–0.4 – 0.3) | 0.09* | |
| Triceps fold V0 [cm] | 2.66 (0.67) | 3.04 (0.80) | 0.07** | |
| Triceps fold V1 [cm] | 2.63 (0.66) | 3.01 (0.83) | 0.07** | |
| Triceps fold V2 [cm] | 2.60 (2.26 – 3.14) | 3.00 (2.54 – 3.34) | 0.34* | |
| Delta Triceps fold [cm] | 0.10 (–0.20 – 0.30) | –0.19 (–0.42 – –0.06) | **0.046*** | |
| Biceps fold V0 [cm] | 1.80 (1.36 – 2.48) | 2.30 (1.40 – 3.14) | 0.32* | |
| Biceps fold V1 [cm] | 1.86 (1.32 – 2.50) | 2.14 (1.74 – 3.00) | 0.31* | |
| Biceps fold V2 [cm] | 2.04 (1.30 – 2.45) | 2.15 (1.55 – 2.95) | 0.31* | |
| Delta Biceps fold [cm] | 0.13 (–0.21 – 0.46) | –0.15 (–0.28 – –0.02) | 0.06* | |
| Scapula fold V0 [cm] | 3.12 (0.88) | 3.22 (0.63) | 0.63** | |
| Scapula fold V1 [cm] | 3.31 (0.92) | 3.14 (0.68) | 0.48** | |
| Scapula fold V2 [cm] | 3.36 (1.06) | 3.14 (0.54) | 0.32$ | |
| Delta Scapula fold [cm] | 0.14 (–0.18 – 0.63) | –0.18 (–0.35 – –0.04) | **0.02*** | |
| Hip fold V0 [cm] | 2.66 (0.59) | 3.13 (0.80) | **0.02**** | |
| Hip fold V1 [cm] | 2.67 (0.67) | 3.01 (0.84) | 0.11** | |
| Hip fold [cm] | 2.70 (2.11 – 3.20) | 3.16 (2.50 – 3.90) | 0.14* | |
| Delta Hip fold [cm] | –0.13 (–0.26 – 0.24) | –0.24 (–0.36 – 0.06) | 0.11* | |

* Mann–Whitney test; ** Student’s t–test $ Welch’s t–test

| Table 4a. | | | | |  |
| --- | --- | --- | --- | --- | --- |
| Tested parameter | Control group  N = 62 | Intervention group <5000 steps  N = 29 | Intervention group >5000 steps  N = 24 | p | |
| Completion of delivery [week] | 34.2 (5.6) | 31.8 (5.1) | 31.7 (5.1) | 0.0497+, a,b | |
| Body weight before pregnancy [kg] | 98 (89 – 113) | 95 (90 – 109) | 111 (101 – 128) | 0.01# b,c | |
| Weight gain during pregnancy [kg] | 4.6 (0.5 – 8.0) | 6.0 (3.0 – 12.0) | –2.7 (–7.4 – –0.75) | <0.001# b,c | |
| Weight gain from V0 to V1 [kg] | 1.0 (–1 – 4.0) | 1.0 (–3.0 – 8.0) | –4.0 (–5.7 – –0.6) | <0.001# b,c | |
| BMI before pregnancy [kg/m^2^] | 1.0 (–1 – 4.0) | 1.0 (–3.0 – 8.0) | –4.0 (–5.7 – –0.6) | <0.001# b,c | |
| BMI gain during pregnancy [kg/m^2^] | 1.0 (–1 – 4.0) | 1.0 (–3.0 – 8.0) | –4.0 (–5.7 – –0.6) | <0.001# b,c | |

+ ANOVA; # Kruskal–Wallis ANOVA; ^ chi^2^–test^;^ $ Yates’ p–value;

Fisher’s POST–HOC a – 1^st^ column vs 2^nd^ column; b – 1 vs 3; c – 2 vs 3 when ANOVA;

DUNN BONFERRONI POST–HOC as Kruskal Wallis ANOVA;

& full–term pregnancies after 37 weeks of gestation were included
